# Essential Medicines Are More Available than Other Medicines around the Globe

**DOI:** 10.1371/journal.pone.0087576

**Published:** 2014-02-12

**Authors:** Yaser T. Bazargani, Margaret Ewen, Anthonius de Boer, Hubert G. M. Leufkens, Aukje K. Mantel-Teeuwisse

**Affiliations:** 1 Division of Pharmacoepidemiology and Clinical Pharmacology, Utrecht Institute for Pharmaceutical Sciences, Utrecht University, Utrecht, the Netherlands; 2 Health Action International-Global, Amsterdam, the Netherlands; Nottingham University, United Kingdom

## Abstract

**Background:**

The World Health Organization (WHO) promotes the development of national Essential Medicines Lists (EMLs) in order to improve the availability and use of medicines considered essential within health care systems. However, despite over 3 decades of international efforts, studies show an inconsistent pattern in the availability of essential medicines. We evaluated and compared the availability of essential medicines, and medicines not included in national EMLs, at global and regional levels.

**Methods:**

Medicine availability in the public and private sector were calculated based on data obtained from national and provincial facility-based surveys undertaken in 23 countries using the WHO/HAI methodology. The medicines were grouped according to their inclusion (‘essential’) or exclusion (termed ‘non-essential’) in each country’s EML current at the time of the survey. Availability was calculated for originator brands, generics and any product type (originator brands or generics) and compared between the two groups. Results were aggregated by WHO regions, World Bank country income groups, a wealth inequality measure, and therapeutic groups.

**Findings:**

Across all sectors and any product type, the median availability of essential medicines was suboptimal at 61·5% (IQR 20·6%–86·7%) but significantly higher than non-essential medicines at 27·3% (IQR 3·6%–70·0%). The median availability of essential medicines was 40·0% in the public sector and 78·1% in the private sector; compared to 6·6% and 57·1% for non-essential medicines respectively. A reverse trend between national income level categories and the availability of essential medicines was identified in the public sector.

**Interpretation:**

EMLs have influenced the provision of medicines and have resulted in higher availability of essential medicines compared to non-essential medicines particularly in the public sector and in low and lower middle income countries. However, the availability of essential medicines, especially in the public sector does not ensure equitable access.

## Introduction

The Essential Medicines List (EML) is promoted by the World Health Organization (WHO) as a means to facilitate equality in access to medicines across the globe. It has been created to satisfy the priority health care needs of societies in terms of availability and affordability of efficacious medicines. [1] Since the introduction of the WHO model list for essential drugs in 1977, whether and to what extent an EML should be implemented has been a challenge to national authorities. However, in accordance with the Universal Declaration of Human Rights [2] and the Millennium Development Goals’ targets [3], the WHO strongly recommends global implementation and regular updates of national EMLs to ensure the availability of essential medicines, in particular in low and middle income countries (LMICs). [4].

According to the WHO, national EMLs exist in 134 countries. Of these, 94% use the EML as a basis for public procurement of medicines, which is the primary source of access to priority medicines particularly for the poor. [5] However, having an EML does not guarantee the availability of essential medicines in health care facilities.

To ensure equitable access and the rational use of medicines and medical technologies, the WHO has set a number of targets in its medium-term strategic plan (2008–2013) one of which is 80% availability of medicines in all sectors. [6] However, recent studies have shown a lack of availability of medicines in LMICs. The availability of a basket of 15 medicines was assessed in the public and private sectors of 36 LMICs by Cameron et al. [7] Overall, generic medicines were not adequately available in both the public and private sectors (median availability 38% and 64%, respectively). However, wide variations were observed in both sectors among the countries. Several studies included assessments of the availability of medicines for acute versus chronic conditions [8], cardiovascular medicines in developing countries [9], anti-epileptics in Zambia [10] and in Laos [11], and anti-diabetic medicines in the Philippines. [12] Similar conclusions were drawn in these studies, namely that the overall availability of widely used medicines including generics was suboptimal, particularly in the public sector. In contrast, some studies showed adequate medicines’ availability. Studies on standardized lists of commonly used medicines in Sudan [13] and Burkina Faso [14] showed a satisfactory level of nearly 80% availability of the surveyed medicines.

None of these studies focused exclusively on essential medicines. The following studies may provide an insight on the extent of availability of medicines included in national EMLs in different countries. Above 75% average availability of generic medicines was reported in a national survey of essential medicines in the private sector in Sri Lanka [15] and in the public sector and popular pharmacies in Brazil [16], as well as in provincial surveys on essential medicines in Ethiopia [17], primary health care centers’ essential medicines in India [18], and the children’s national rural health mission list in the public sector in India. [19] Conversely, a national study on the availability of pediatric essential medicines in Sri Lanka’s public sector [20] as well as provincial surveys on national essential medicines in China [21], [22] all pointed to inadequate availability of the essential medicines studied.

In view of the limited number of studies to date, no extensive conclusions can be drawn on whether essential medicines are indeed adequately available as a result of global efforts to implement essential medicines policies. Therefore, this international study was undertaken to assess differences in the availability of essential medicines compared to medicines which are not on EMLs (hereafter called “non-essential medicines”) in both public and private sectors.

## Methods

### Data Source

Data on the availability of individual medicines at facility level were obtained from the WHO/Health Action International (HAI) database of medicine prices, availability, affordability and price components. [23] The database consists of findings from facility-based surveys on the availability and affordability of selected medicines - either generics or originator brands - in the public and the private sectors of numerous LMICs. All surveys have been conducted in compliance with a standard methodology developed by WHO/HAI. [24], [25] The survey medicines comprised a core list and a supplementary list of medicines. The core list was selected from medicines indicated for common chronic or acute conditions in global and regional scales. The supplementary list included medicines selected at country level based on their local importance for national health care systems. Practical details of the surveys as well as the validation method have been explained elsewhere. [7], [23], [26].

As a second step, all national EMLs were obtained from the WHO database of essential medicine lists and formularies which is freely accessible on the web. [27].

### Study Inclusion

All surveys included in the WHO/HAI database on 15 April 2012 were included in our study except (a) those for which the national EML, current at the time of the survey time, was not available, and (b) eight pilot surveys where availability was assessed using a different methodology. Where multiple surveys were conducted in a country, average availability was calculated without weighting. Table 1 lists the 23 countries (28 surveys) included in the study.

**Table 1:** Countries and surveys included in the study.

Country	Survey year	Number of facilitiessurveyed	Number of essentialmedicines surveyed/Totalno. of surveyed medicines	WHO region	WB income group*	Gini Index**	Gini Index (year)
Bolivia	2008	60	42/50	Americas	LMIC	56.29	2008
Brazil	2008	56	43/50	Americas	UMIC	55.07	2008
Cameroon	2005	60	25/36	Africa	LIC	38.91	2007
Chad	2004	43	18/22	Africa	LIC	39.78	2003
China	2010	86	33/47	Western Pacific	UMIC	42.48	2005
Congo, Rep.	2007	58	28/32	Africa	LMIC	47.32	2005
Ethiopia	2004	87	44/47	Africa	LIC	29.83	2005
Ghana	2004	112	36/49	Africa	LIC	42.76	2006
Indonesia	2010	153	43/50	South-East Asia	LMIC	34.01	2005
Kenya	2004	157	33/45	Africa	LIC	47.68	2005
Malaysia	2004	72	23/47	Western Pacific	UMIC	37.91	2004
Mali	2004	64	35/37	Africa	LIC	38.99	2006
Mexico	2009	28	38/42	Americas	UMIC	48.28	2008
Nicaragua	2008	105	30/43	Americas	LMIC	40.47	2005
Nigeria	2004	124	27/29	Africa	LIC	42.93	2004
Pakistan	2004	78	20/29	Eastern Mediterranean	LIC	31.18	2005
South Africa	2004	45	35/42	Africa	UMIC	67.4	2006
Tajikistan	2005	40	30/34	Europe	LIC	33.61	2004
Tanzania	2004	111	29/44	Africa	LIC	37.58	2007
Thailand	2006	41	40/43	South-East Asia	LMIC	42.35	2006
Uganda	2004	60	32/45	Africa	LIC	42.62	2006
Yemen	2006	40	17/35	Eastern Mediterranean	LIC	37.69	2005
India	2003–2011	656	185/232	South-East Asia	LIC†	33.38	2005

*As at the time of the survey. LIC: low income country, LMIC: lower middle income country, UMIC: upper middle income country.

**Data is closest to the year of the survey.

† 2003–2005.

### Data Analysis

Data were analyzed at a global scale first. Pooled data of the studied medicines were assigned to either essential or non-essential medicines, based on the national EML at the time of the survey. Availability was calculated as the percentage of medicine outlets surveyed where the medicine was available on the day of data collection. In addition to studying the availability of originator brands and generic equivalents, the availability of any product type (originator brand or generic combined, see Table 2 for definitions) was assessed to determine the overall availability of each medicine.

**Table 2:** Definitions of the technical terms.

Term	Definition
Originator brand medicine	Generally the product that was first authorized worldwide for marketing (normally as a patented product) on the basis of the documentation of its efficacy, safety and quality, according to requirements at the time of authorization. The originator product always has a brand name; this name may, however, vary between countries.^a^
Generic medicine	A pharmaceutical product usually intended to be interchangeable with the originator brand product, manufactured without a license from the originator manufacturer and marketed after the expiry of patent or other exclusivity rights. Generic medicines are marketed either under a nonproprietary name (INN), rather than under a proprietary or brand name. However, they are also quite frequently marketed under brand names, often called “branded generics”.^a^
Patent	A title granted by public authorities that confers a temporary monopoly for the exploitation of an invention upon the person who reveals it, furnishes a sufficiently clear and full description of it, and claims this monopoly.^a^
Data exclusivity	Exclusivity differs from patent protection in that it provides statutory exclusion of others from marketing or use of originator’s test data for subsequent drug applications. Exclusivity terms can run concurrently or in seriatim.^b^

a Health Action International (HAI): http://www.haiweb.org/medicineprices/manual/mp2008/NPrices_Glossary.pdf.

b Mackey TK, Liang BA. Patent and exclusivity status of essential medicines for non-communicable disease. *PLoS One* 2012;7(11):e51022.

The median availability of originator brands, generics and any product type was calculated and compared for essential and non-essential medicines, across all sectors and individual sectors (public, private).

Data were also stratified by World Bank country income groups [28], Gini coefficient as a measure of inequality in wealth [29], and WHO regions. [30] To study the availability gap across different therapeutic groups, data were analyzed according to the Anatomical Therapeutic Chemical (ATC) classifications. [31].

Non-parametric rank tests performed were (1) the Mann–Whitney U test to compare the availability of essential versus non-essential medicines when homogeneity of variances was established, (2) the Moods median test to compare availability of essential versus non-essential medicines when homogeneity of variances was not assumed, and (3) a Kruskal-Wallis one-way analysis of variance by ranks for comparing across World Bank income groups in terms of the availability of essential medicines. To assess if availability gap of essential and non-essential medicines is associated with the Gini coefficient, linear regression analysis was examined. A p-value smaller than 0.05 was considered significant for all the tests.

## Results

### Overall Availability of Medicines

A total of 28 surveys corresponding to 1130 medicines (886 essential medicines) and 2290 facilities were analyzed. As shown in Table 3, the overall median availability of essential medicines for any product type was 61·5% while the availability of non-essential medicines was 27·3%. This difference in availability was driven by generic medicines; the median availability of generic essential medicines was 53·3% versus 19·2% for non-essential generics. The overall median availability of originator brands was 0%, with no significant difference between essential (IQR 0%–18.4%) and non-essential medicines (IQR 0%–21.1%) (p = 0·579). Similar patterns were observed in each sector.

**Table 3:** Median availability of surveyed medicines.

Sector	Product types	Median (IQR) availability ofessential medicines	Median (IQR) availability ofnon-essential medicines	p-value
All sectors	Originator Brand	0.0% (0.0%–18.4%)	0.0% (0.0%–21.1%)	0.579
	Generic	53.3% (15.0%–83.3%)	19.2% (0.0%–64.8%)	0.000
	Any type	61.5% (20.6%–86.7%)	27.3% (3.6%–70.0%)	0.000
Public sector	Originator Brand	0.0%(0.0%–0.0%)	0.0% (0.0%–2.0%)	0.050
	Generic	35.0% (9.1%–73.8%)	5.0% (0.0%–25.0%)	0.000
	Any type	40.0% (10.0%–75.8%)	6.6% (0.0%–30.0%)	0.000
Private sector	Originator Brand	20.0% (0.0%–55.0%)	19.5% (0.0%–50.0%)	0.916
	Generic	66.7% (30.8%–86.7%)	47.4% (6.7%–81.4%)	0.000
	Any type	78.1% (48.2%–91.6%)	57.1% (27.3%–87.5%)	0.000
Other sectors	Originator Brand	0.0% (0.0%–6.7%)	0.0% (0.0%–6.7%)	0.787
	Generic	50.0% (13.2%–82.6%)	20.0% (22.7%–53.1%)	0.000
	Any type	53.3% (15.0%–84.1%)	22.2% (4.4%–56.0%)	0.000

IQR = interquartile range.

### Availability of Medicines in the Public Sector

The median availability of essential and non-essential medicines was 40·0% and 6·6% respectively for any product type. The availability of essential generic medicines in this sector was 35·0% whereas the availability of non-essential generics was 5·0% (p<0·001). Few originator brands were found in the public sector.

### Availability of Medicines in the Private Sector

Availability of any product type was higher in the private sector at a median of 78·1% and 57·1% for essential and non-essential medicines respectively (p<0·001). Likewise, the availability of generics was higher (66·7% and 47·4% for median availability of essential and non-essential medicines respectively) compared to the public sector. The availability of originator brands was similar for essential and non-essential medicines (median availability was 20·0% and 19·5% respectively).

### Availability of Medicines across Income Groups

**Figure 1:**
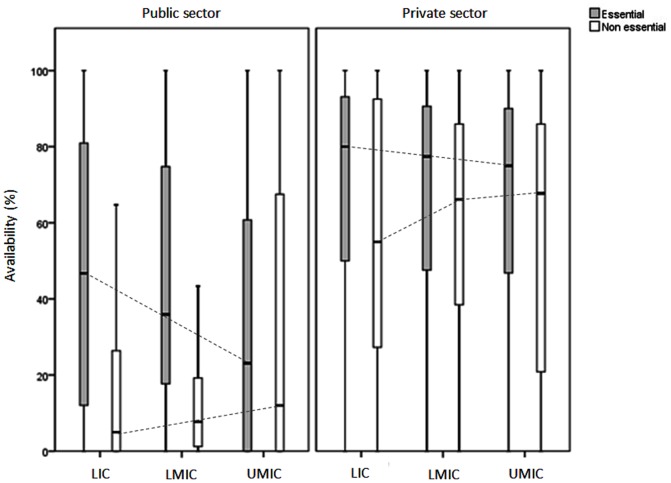
Availability (median, interquartile range, maximum-minimum) of any product type (originator brand/generic) of essential and non-essential medicines by World Bank income level*. * LIC: Low income countries, LMIC: Lower-middle income countries, UMIC: Upper-middle income countries.

In the public sector, a reverse trend (p<0·001) between income level and the availability of essential medicines (any product type) was observed (see Figure 1). As income level increased, the median availability declined. However, the availability did not exceed 50% in any income group. In the private sector, the median availability of essential medicines (any product type) was higher in all income groups ranging from 75% to 80%. In particular, in upper-middle income countries the availability of originator brands was considerably higher than other income groups both for essential and non-essential medicines (40% availability in both groups). Median availability of the two groups of medicines (essential vs. non-essential) differed significantly in low and lower-middle income countries for any product type of medicines (Difference = 25% and 11·3% respectively; p<0·05).

### Availability of Medicines across Geographic Regions

When different WHO regions were taken into account, a significant difference was observed in the availability of any product type between essential medicines versus non-essential medicines in Africa, the Americas and South-East Asia (mean difference of median availability was 41% for the three regions versus 3·6% for the other regions), mainly due to a gap in the availability of generics (37·4% versus 3·3%). The median availability of essential medicines (any product type) was lower than 50% across all WHO regions in the public sector except Europe, although essential medicines were more available than non-essential medicines (mean difference 32·4% for any product type and 36·6% for generics, respectively). In the private sector, the median availability of essential medicines (any product type) ranged from 68·8% to 86·7% across all regions. However, the median availability of non-essential medicines (any product type) was also above 50% across all regions.

### Availability of Medicines across Therapeutic Groups

**Figure 2:**
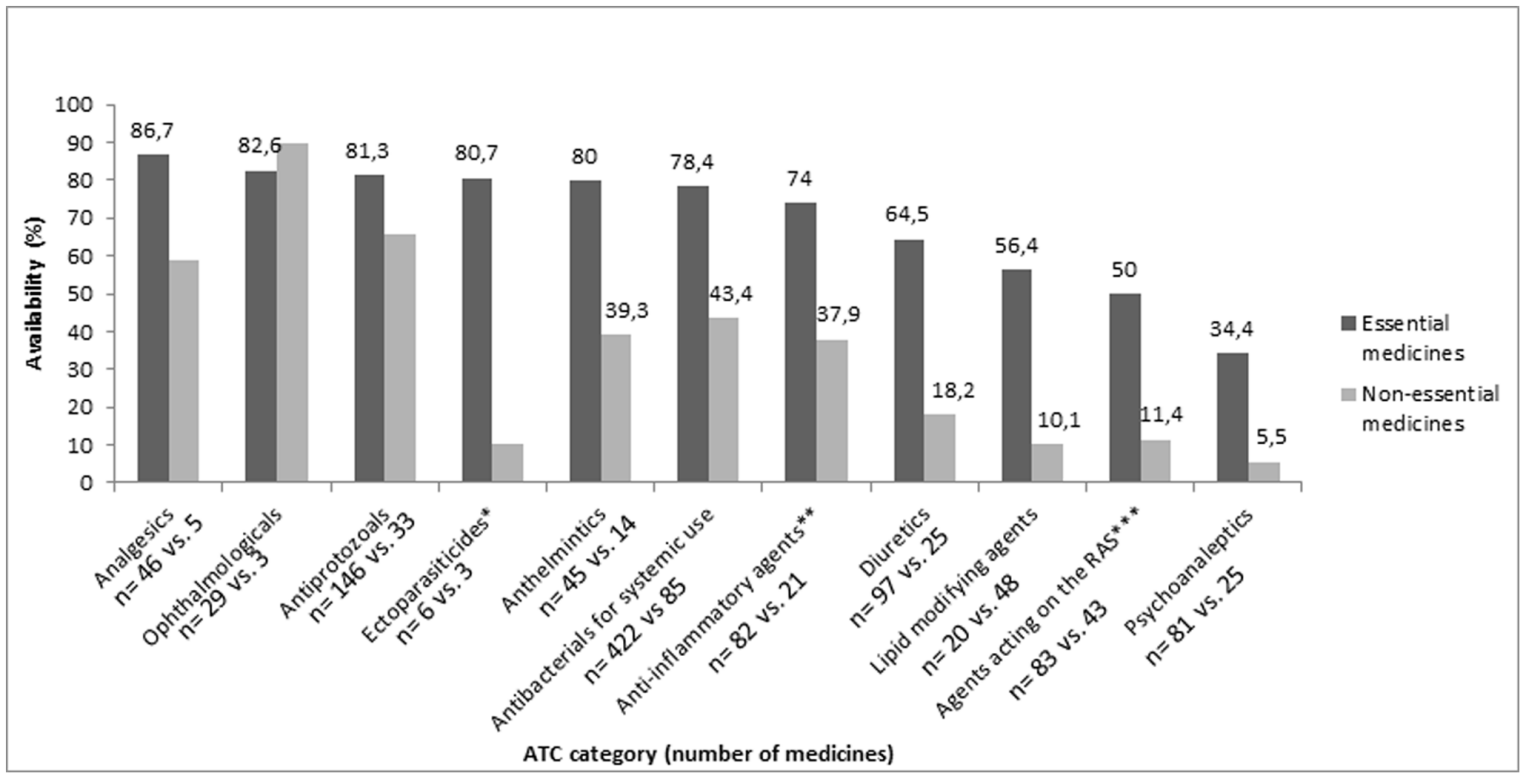
Median availability of any product type (originator brand/generic) of essential and non-essential medicines by ATC categories. *Ectoparasiticides, including scabicides, insecticides and repellents, **Anti-inflammatory and anti-rheumatic products, ***Agents acting on the renin-angiotensin system.

As shown in Figure 2, the median availability of essential antibacterial agents for systemic use, anti-inflammatory and antirheumatic medicines, analgesics, antiprotozoals, anthelmintics, and ophthalmologicals was reasonably high ranging from 74·0% to 86·7% for any product type, across all sectors. For diuretics, agents acting on the renin-angiotensin system, lipid modifying agents, antibacterial agents for systemic use, and psychoanaleptics essential medicines were more available than non-essential medicines (average median difference = 39%) for any product type.

No relationship was identified between the availability gap of essential versus non-essential medicines and the Gini coefficient in an overall view as well as in both the public and the private sectors (regression coefficients <0·005 and p>0·50). The level of income disparity was not able to explain observed variation of the availability gap. (R^2^ = 0·000, 0·014 and 0·008, respectively).

## Discussion

According to our study essential medicines were more available than non-essential medicines. The median availability of essential medicines was 61.5%, of which a substantial contribution was made by generic medicines (53·3%). The results showed considerable variation in availability of essential medicines across WHO regions, national income levels, and therapeutic groups.

Median availability of any product type of essential medicines in the public sector was 40% in our study which is extremely suboptimal. However, in this sector, essential medicines were substantially more available than non-essential medicines. This may indicate the preferential attention of governments towards essential medicine supply in this sector as procurement and supply chain management is often directly under their supervision and control. [32] Only few originator brands of essential medicines were found in this sector which may reflect procurement efficiencies given price differentials between originator brands and generics. [33], [34] The suboptimal availability of essential medicines in the public sector was observed in all WHO regions.

Median availability of essential medicines was higher in the private sector compared to the public sector, being just under the WHO’s benchmark target. The difference in availability between essential and non-essential medicines was smaller in the private sector (compared to the public sector), suggesting a less regulated, less prioritized and closer to free market environment. Unlike the public sector where generics greatly predominate, in the private sector originator brands increasingly contributed to the overall availability of essential medicines. While prioritization of essential medicines has been mainly intended for public sector procurement, the private sector seems to have been affected as well. The poor availability of essential medicines in the public sector may be an explanation for this phenomenon. As a consequence, patients are forced into the private sector to access medicines. This would result in an increased demand for essential medicines in the private sector which might lead to increased supply. This shift is usually associated with an increased financial burden for patients which may result in undertreatment, particularly for chronic diseases in LMICs. [8].

The findings of this study suggest that essential medicines policies have been more successfully implemented in the public sectors of low- and lower-middle income countries than in upper-middle income countries. The inverse trend observed between national income level categories and availability of essential medicines might confirm that as resources get scarcer, prioritization becomes a more crucial necessity. Moreover, our findings indicate a higher reliance on the private sector availability of (essential) medicines with an increase in national income level category.

Cameron *et al* have shown that across 40 LMICs the availability of medicines to treat chronic diseases was less than those for acute conditions. [8] In our study, cardiovascular (antihypertensive and lipid modifying agents) and psychoanaleptic medicines were among the therapeutic groups for which the availability of essential medicines was far greater than non-essential medicines. This may indicate that despite lower availability of medicines for chronic conditions, some efforts have been taken to make essential chronic disease medicines more available in the public and private sector of LMICs. Similarities and differences in the way that decisions regarding the selection of essential medicines are taken across countries should be investigated. For example, the extent to which evidence-based decision making is practiced, consistency of choices with local treatment guidelines, and the influence of regional epidemiology and burden of disease.

The WHO/HAI methodology has been validated and over the last decade has been considered the standard for measuring medicine availability, price, affordability and price components. Some general limitations of the WHO/HAI methodology concerning the quality of surveyed medicines, diversity in level of care in facilities within a survey and its cross-sectional design instead of overtime measurements (panel survey) have been discussed elsewhere. [7], [8] The small number of countries per region included in the dataset (except for Africa) makes conclusions difficult to extend to each entire region. Therefore drawing general conclusions regarding the regions has been avoided.

The time interval between the implementation of a national EML and conducting the WHO/HAI survey was not consistent between the countries. It ranged from a couple of months to over two or three years. However, the EML to which the medicines were compared was the current list at the time of survey. The limited number of non-essential surveyed medicines (on average 11 medicines per survey; SD = 10) compared to the essential medicines (on average 39 medicines per survey; SD = 33) raises concerns regarding comparability of the two groups of medicines. This limitation is partly due to the fact that inclusion of essential medicines in WHO/HAI surveys is encouraged. [24] Nevertheless, this study is the most comprehensive analysis to date to assess the availability of essential medicines at a global scale. Endeavors towards prioritization have made essential medicines more available among enormous number of medicines launched to the market and therefore contributes to more efficient allocation of limited health care financial resources.

Low availability of essential medicines, especially in the public sector, needs more attention and efforts by the relevant authorities to ensure these medicines are stocked in facilities. Employing supply chain management (SCM) techniques might improve the situation. Pooled procurement at national or regional levels in order to benefit from economy of scale and monopsony is an example. However, sustainability of such joint actions requires some prerequisites such as commitment of all stakeholders and a high level of harmonization. Regional collaborations to share procurement information (e.g. prices and rebates) can be seen as a first practical step. Effective inventory management, basing order quantification on accurate estimates of actual need, long term supply contracts and constantly keeping track of procurement performance are other SCM techniques which might ultimately improve the availability. [32], [35].

Sufficient sustainable financing is a critical factor in ensuring access to essential medicines in the long run. However, short to mid-term interventions can also improve access. Over recent years international donors have funded programs which have saved the lives of millions of people in LMICs. [36] Such programs can benefit societies most if they are aligned with national health care priorities. It has been argued that donors do not necessarily fund the most relevant interventions. [37] Procurement necessities of each individual country might be best reflected in its essential medicines list.

Enhancing local manufacturing capacities, and using flexibilities in international trade agreements such as TRIPS for patented products, to improve the availability of essential medicines at more affordable prices should not be ignored by the authorities. Chen et *al* described a case in which local manufacturers did not produce more than 60% of the essential medicines they were licensed to supply. [21] It has been stated that local production has the potential to improve access to medicines. [38] However, there are some doubts regarding the existence of enough empirical supportive evidence. [39] Mackey et *al* did not find valid patents or data exclusivity provisions for the medicines listed for non-communicable disease in the WHO’s model list of essential medicines. [40] Nevertheless, patent protection is not an exclusion criteria for selection of medicines for the WHO’s model list. Besides patented medicines can be found in national EMLs as well.

The WHO prequalification program for medicines (including HIV/AIDS, tuberculosis, antimalarial and reproductive medicines as well as vaccines) has provided further opportunities for the procurement of quality-assured medicines in a competitive and affordable manner. [41], [42] It would be favorable if such programs could be extended to all ranges of essential medicines.

As far as the private sector is concerned, even though reasonably high availability of essential medicines can be positively interpreted, a great deal of attention should be paid to affordability concerns. The noticeable contribution of originator brand products to overall availability and extremely high prices of medicines compared to international reference prices [7] indicates the value of moving towards implementation of universal reimbursement systems that ensure equitable access to medicines for the entire society regardless of sectorial concerns.

## Conclusions

This study shows that essential medicines are more available in health care facilities than non-essential medicines. It can be concluded that implementation of EMLs has led to prioritization of essential medicines, especially in low- and lower-middle income countries. It seems that this policy intervention (introduction and promotion of EMLs) has been moderately effective over the past couple of years.

However, the availability of essential medicines is still far from ideal, in particular in the public sector, suggesting that sustainable adequate funding in parallel with employing supply chain management techniques is crucial to ensure access of patients to needed medicines.

## References

[1] WHO (2012) Health topics/Essential medicines. Available: http://www.who.int/topics/essential_medicines/en/Accessed 2012 Dec 2.

[2] UN, Universal declaration of human rights, article 25: “(1) everyone has the right to a standard of living adequate for the health and well-being of himself and of his family, including food, clothing, housing and medical care…”. Available: http://www.un.org/en/documents/udhr/index.shtml#a25. Accessed 2012 Dec 2.

[3] UN, Millennium development goals, MDG8: Develop a global partnership for development, Target8E: “In cooperation with pharmaceutical companies, provide access to affordable essential medicines in developing countries”. Available: http://www.un.org/millenniumgoals/global.shtml. Accessed 2012 Dec 2.

[4] WHO (2012) 20 ways the world health organization helps countries reach the millennium development goals. Available: http://who.int/topics/millennium_development_goals/20ways_mdgs_20100517_en.pdf. Accessed 2012 Dec 2.

[5] van den Ham R, Bero L, Laing R (2011) The worlds medicines situation 2011, selection of essential medicines. Available: http://apps.who.int/medicinedocs/documents/s18770en/s18770en.pdf. Accessed 2012 Dec 2.

[6] WHO (2008) WHO MEDIUM-TERM STRATEGIC PLAN 2008–2013; plan M-TS. 2008–2013. Available: http://apps.who.int/gb/e/e_amtsp.html. Accessed 2012 Dec 2.

[7] Cameron A, Ewen M, Ross-Degnan D, Ball D, Laing R (2009) Medicine prices, availability, and affordability in 36 developing and middle-income countries: A secondary analysis. Lancet 373: 240–249 10.1016/S0140-6736(08)61762-6 19042012

[8] Cameron A, Roubos I, Ewen M, Mantel-Teeuwisse AK, Leufkens HG, et al (2011) Differences in the availability of medicines for chronic and acute conditions in the public and private sectors of developing countries. Bull World Health Organ 89: 412–421 10.2471/BLT.10.084327;10.2471/BLT.10.084327 21673857 PMC3099556

[9] van Mourik MS, Cameron A, Ewen M, Laing RO (2010) Availability, price and affordability of cardiovascular medicines: A comparison across 36 countries using WHO/HAI data. BMC Cardiovasc Disord 10: 25 10.1186/1471-2261-10-25 20534118 PMC2898673

[10] Chomba EN, Haworth A, Mbewe E, Atadzhanov M, Ndubani P, et al (2010) The current availability of antiepileptic drugs in zambia: Implications for the ILAE/WHO “out of the shadows” campaign. Am J Trop Med Hyg 83: 571–574 10.4269/ajtmh.2010.10-0100 20810822 PMC2929053

[11] Odermatt P, Ly S, Simmala C, Angerth T, Phongsamouth V, et al (2007) Availability and costs of antiepileptic drugs and quality of phenobarbital in vientiane municipality, lao PDR. Neuroepidemiology 28: 169–174 10.1159/000103270 17536229

[12] Higuchi M (2010) Access to diabetes care and medicines in the philippines. Asia Pac J Public Health 22: 96S–102S 10.1177/1010539510373005 20566540

[13] Cheraghali AM, Idries AM (2009) Availability, affordability, and prescribing pattern of medicines in sudan. Pharm World Sci 31: 209–215 10.1007/s11096-009-9282-3 19263235

[14] Saouadogo H (2011) Measuring availability, affordability and management of essential medicines in public hospitals of burkina faso. World Hosp Health Serv 47: 8–11. 21675631

[15] Senarathna SM, Mannapperuma U, Fernandopulle BM (2011) Medicine prices, availability and affordability in sri lanka. Indian J Pharmacol 43: 60–63 10.4103/0253-7613.75672 21455424 PMC3062124

[16] Bertoldi AD, Helfer AP, Camargo AL, Tavares NU, Kanavos P (2012) Is the brazilian pharmaceutical policy ensuring population access to essential medicines? Global Health 8: 6–8603-8-6 10.1186/1744-8603-8-6;10.1186/1744-8603-8-6 22436555 PMC3511298

[17] Carasso BS, Lagarde M, Tesfaye A, Palmer N (2009) Availability of essential medicines in ethiopia: An efficiency-equity trade-off? Trop Med Int Health 14: 1394–1400 10.1111/j.1365-3156.2009.02383.x 19754520

[18] Dixit R, Vinay M, Jayasree T, Ubedulla S, Manohar VS, et al (2011) Availability of essential medicines: A primary health care perspective. Indian J Pharmacol 43: 599–600 10.4103/0253-7613.84981 22022009 PMC3195136

[19] Gitanjali B, Manikandan S (2011) Availability of five essential medicines for children in public health facilities in india: A snapshot survey. J Pharmacol Pharmacother 2: 95–99 10.4103/0976-500X.81900 21772768 PMC3127358

[20] Balasubramaniam R, Beneragama BV, Sri Ranganathan S (2011) A national survey of availability of key essential medicines for children in sri lanka. Ceylon Med J 56: 101–107. 22164746 10.4038/cmj.v56i3.3597

[21] Chen W, Tang S, Sun J, Ross-Degnan D, Wagner AK (2010) Availability and use of essential medicines in china: Manufacturing, supply, and prescribing in shandong and gansu provinces. BMC Health Serv Res 10: 211 10.1186/1472-6963-10-211 20637116 PMC2915989

[22] Yang H, Dib HH, Zhu M, Qi G, Zhang X (2010) Prices, availability and affordability of essential medicines in rural areas of hubei province, china. Health Policy Plan 25: 219–229 10.1093/heapol/czp056 19955093

[23] Health Action International (2012) WHO/HAI project on medicine prices and availability. Available: http://www.haiweb.org/MedPriceDatabase. Accessed 2012 April 2.

[24] Health Action International and WHO (2008) Measuring medicines prices,availability, affordability and price components, 2nd edition. Available: http://www.who.int/medicines/areas/access/OMS_Medicine_prices.pdf. Accessed 2012 April 2.

[25] Health Action International and WHO (2003) Medicine prices: A new approach to measurement,2003 edition. working draft for field testing and revision. Available: http://whqlibdoc.who.int/hq/2003/WHO_EDM_PAR_2003.2.pdf. Accessed 2012 April 2.

[26] Madden JM, Meza E, Ewen M, Laing RO, Stephens P, et al (2010) Measuring medicine prices in peru: Validation of key aspects of WHO/HAI survey methodology. Rev Panam Salud Publica 27: 291–299. 20512232 10.1590/s1020-49892010000400008

[27] WHO (2012) WHO database for national medicines List/Formulary/Standard treatment guidelines. Available: http://www.who.int/selection_medicines/country_lists/en/Accessed 2012 April 15.

[28] World Bank (2011) Country classification. Available: http://data.worldbank.org/about/country-classifications. Accessed 2012 April 15.

[29] World Bank (2011) GINI index. Available: http://data.worldbank.org/indicator/SI.POV.GINI. Accessed 2012 April 15.

[30] WHO (2012) WHO regions and the location of the regional offices. Available: http://www.who.int/about/regions/en/Accessed 2012 April 15.

[31] Norwegian Institute of Public Health, WHO Collaborating Centre for Drug Statistics Methodology (2012) Anatomical Therapeutic Chemical (ATC) classification system for medicines. Available: http://www.whocc.no/atc_ddd_index/Accessed 2012 April 15.

[32] Management Sciences for Health (2012) Managing access to medicines and health technologies. Arlington, VA: Management Sciences for Health.

[33] Cameron A, Mantel-Teeuwisse AK, Leufkens HG, Laing RO (2012) Switching from originator brand medicines to generic equivalents in selected developing countries: How much could be saved? Value Health 15: 664–673 10.1016/j.jval.2012.04.004 ; 10.1016/j.jval.2012.04.004 10.1016/j.jval.2012.04.004; 10.1016/j.jval.2012.04.004 22867775

[34] Volger S (2012) How large are the differences between originator and generic prices? analysis of five molecules in 16 european countries. Farmeconomia Health economics and therapeutic pathways 13: 29–41.

[35] Kanavos P, Das P, Durairraj V, Laing R, Abegunde DO (2011) The worlds medicines situation 2011, Options for financing and optimizing medicines in resource-poor countries. Available: http://apps.who.int/medicinedocs/documents/s20033en/s20033en.pdf Accessed 2012 Dec 2.

[36] Voelker R (2010) One casualty of global economic crisis: Uncertain finances for HIV/AIDS programs. JAMA 304: 259–261 10.1001/jama.2010.962 20639554

[37] Twisselmann B (2005) Learning from low income countries. BMJ 330: 479.

[38] WHO (2011) Local production for access to medical products: Developing a framework to improve public health. Available: http://www.who.int/phi/publications/Local_Production_Policy_Framework.pdf Accessed: 2012 Dec 5.

[39] Kaplan WA, Ritz LS, Vitello M (2011) Local production of medical technologies and its effect on access in low and middle income countries: A systematic review of the literature. South Med Rev 4: 51–61 10.5655/smr.v4i2.1002 ; 10.5655/smr.v4i2.1002 10.5655/smr.v4i2.1002; 10.5655/smr.v4i2.1002 23093883 PMC3471180

[40] Mackey TK, Liang BA (2012) Patent and exclusivity status of essential medicines for non-communicable disease. PLoS One 7: e51022 10.1371/journal.pone.0051022 ; 10.1371/journal.pone.0051022 10.1371/journal.pone.0051022; 10.1371/journal.pone.0051022 23226453 PMC3511406

[41] Robertson J, Hill SR (2007) The essential medicines list for a global patient population. Clin Pharmacol Ther 82: 498–500 10.1038/sj.clpt.6100392 17952104

[42] Chakma J, Masum H, Perampaladas K, Heys J, Singer PA (2011) Indian vaccine innovation: The case of shantha biotechnics. Global Health 7: 9 10.1186/1744-8603-7-9 21507259 PMC3110116

